# Genetic Variants of Thymic Stromal Lymphopoietin and Non-IgE-Mediated Food Allergy

**DOI:** 10.3390/ijms27104490

**Published:** 2026-05-17

**Authors:** Maria-Teodora Coșoreanu, Felicia Galoș, Luis-Ovidiu Popa, Camelia-Elena Berghea, Mara-Ioana Ionescu, Andreea Ioan, Mara-Ingrid Rieber, Eliza-Elena Cinteză, Olivia-Mihaela Popa

**Affiliations:** 1 Department of Pediatrics, Carol Davila University of Medicine and Pharmacy, 020021 Bucharest, Romania; maria-teodora.ilie@drd.umfcd.ro (M.-T.C.); camelia.berghea@umfcd.ro (C.-E.B.); andreea.berariu@drd.umfcd.ro (A.I.); eliza.cinteza@umfcd.ro (E.-E.C.); 2 Department of Pediatrics, Marie Curie Emergency Children’s Hospital, 041451 Bucharest, Romania; mara-ioana.ionescu@umfcd.ro; 3 Molecular Biology Department, Grigore Antipa National Museum of Natural History, 011341 Bucharest, Romania; 4 Department of Physiology-Neuroscience, Carol Davila University of Medicine and Pharmacy, 020021 Bucharest, Romania; 5 Department of Clinical Immunology, National Institute for Mother and Child Health Alessandrescu-Rusescu, 020395 Bucharest, Romania; 6 Department of Pathophysiology and Immunology, Carol Davila University of Medicine and Pharmacy, 020021 Bucharest, Romania; mara-ingrid.rieber@rez.umfcd.ro (M.-I.R.); olivia.popa@umfcd.ro (O.-M.P.)

**Keywords:** non-IgE-mediated food allergies, TSLP, gene polymorphisms, rs10062929, rs3806933, atopic dermatitis

## Abstract

Representing a pathology frequently encountered in the clinical pediatric practice, non-IgE-mediated food allergy is characterized by delayed immune-mediated adverse reactions to foods. Various genetic and environmental factors play complex and incompletely elucidated roles in the development of this type of allergic pathology. In the present research, we aimed to assess the associations of the *TSLP* gene single-nucleotide polymorphisms (SNPs) rs3806933 and rs10062929 with the risk of non-IgE-mediated food allergy, as well as with other characteristics of these patients. The study included 87 Romanian patients with non-IgE-mediated food allergy and 115 healthy controls. The two SNPs were genotyped using Real-Time PCR TaqMan Allelic Discrimination Assays. No associations were identified between rs3806933 or rs10062929 and the risk of non-IgE-mediated food allergy. The frequency of the minor allele C of rs3806933 was lower in children with atopic dermatitis (35.4% vs. 46.8%), a positive family history of atopic disease (39.0% vs. 47.8%), and elevated levels of total IgE (39.2% vs. 45.7%), but not statistically significant.

## 1. Introduction

Non-IgE-mediated food allergies represent adverse reactions to food proteins with a pathophysiology involving immunologic mechanisms other than systemic immunoglobulin E (IgE) production. They are characterized by a delayed onset after ingestion of the trigger food, with the main clinical manifestations being gastrointestinal [1,2,3]. Non-IgE-mediated food allergies are a heterogeneous group encompassing several clinical entities with overlapping symptoms: food protein-induced enterocolitis syndrome (FPIES), food protein-induced allergic proctocolitis (FPIAP), food protein-induced enteropathy (FPE), Eosinophilic esophagitis (EoE), food protein-induced gastroesophageal reflux disease (FPGORD), and food protein-induced constipation (FPC) [1,4].

Over the last few decades, an increase in the prevalence of non-IgE-mediated food allergies has been described [1]. The interplay between genetic and environmental factors leads to a higher risk of developing the disease, but the specific underlying mechanisms are not elucidated [5,6]. Multiple studies have reported the association between a positive family history of atopy and an increased risk of food allergy, thus supporting the existence of a strong genetic background [7,8,9,10]. The exact pathogenic mechanisms leading to the development of non-IgE-mediated food allergies are incompletely understood [3]. The cellular component of the immune system plays the most important role, involving the activation of the T-cell pathways and innate immunity processes with the production of specific cytokines [11].

The most common trigger of non-IgE-mediated food allergy in children is cow’s milk [1,3]. The majority of the clinical manifestations are gastrointestinal and include food refusal, dysphagia, vomiting, diarrhea, hematochezia, constipation, anal fissures, etc. [12]. General (failure to thrive, iron deficiency), cutaneous (atopic dermatitis), and respiratory manifestations (rhinitis, chronic cough) can also be associated [12]. Although the prognosis is favorable for most children, the elimination diet has an important impact on the quality of life of patients and their families [3,13]. Moreover, in severe forms of the disease, emergency treatment is needed, as life-threatening manifestations such as shock can be present [13].

Thymic stromal lymphopoietin (TSLP) is a pleiotropic cytokine with a key role in skewing the immune response toward the T-helper 2 (Th2) phenotype [14]. TSLP is constitutively expressed in the epithelial cells of the gastrointestinal tract, skin, and lungs; at the same time, other cytokines, mechanical stimuli, and noxious substances can also trigger TSLP production in various cell types such as fibroblasts, dendritic cells, mast cells, and airway smooth muscle cells [15,16,17]. The impaired integrity of the epithelial barrier represents a key feature of non-IgE-mediated food allergy, and TSLP represents one of the first cytokines expressed by the activated epithelial cells upon the breach of the mucosal barrier [18,19]. TSLP, along with other cytokines (such as IL-33 and IL-25), plays a critical role in type 2 allergic responses at the barrier levels, being involved in triggering an immune response to external factors, and also in regulating tissue restoration and epithelial barrier repair after injury [20]. The absence of an intact epithelial barrier and local inflammatory processes leads to an increased antigen passage and, therefore, to additional allergic reactions to the antigens, in a continuous self-amplifying process [14].

TSLP can induce the activation of dendritic cells, which determines Th2 differentiation of naïve T cells and is also able to directly promote naïve T cells to Th2 cells [14]. Moreover, it was found that TSLP can influence the activity of B cells, eosinophils, basophils, mast cells, NK cells, smooth muscle cells, and tumor cells [16,21,22,23].

Accumulating data demonstrate the role of TSLP in the pathogenesis of allergic diseases, including EoE, atopic dermatitis, asthma, and allergic rhinoconjunctivitis [14,16,17,21,24,25]. It was found that TSLP was absent in the normal skin areas of patients with atopic dermatitis, but it was highly expressed in atopic dermatitis lesions [14]. In pediatric patients, increased TSLP expression in esophageal biopsies was reported in subjects with active EoE compared with those with inactive EoE and controls [22,26]. These findings are supported by the promising results of clinical research assessing the efficacy and safety of Tezepelumab, an anti-TSLP human monoclonal antibody, for the treatment of severe forms of EoE, while the drug is already approved for the treatment of asthma [27,28,29].

The *TSLP* gene, located on chromosome 5q22.1, has four exons and encodes two transcript variants: a long (lfTSLP) and a short (sfTSLP) TSLP isoform [29,30,31]. The two variants are generated by using different initiation methionine codons during the protein translation process [32]. The long isoform of TSLP is produced in inflammatory contexts, while the short isoform is expressed in steady conditions [33]. Several studies, including genome-wide association studies (GWASs), have explored the associations between single nucleotide polymorphisms (SNPs) of this gene (rs3806933, rs3806932, rs10062929, rs1837253, rs2289276, rs2289277, rs2289278, rs1898671) and EoE [24,25,34,35,36], IgE-mediated food allergy [35,37], atopic dermatitis [21,30,37], and asthma [38,39]. Among these polymorphisms, the rs3806933 (-847C/T) SNP is located in the promoter region of lfTSLP and leads to increased binding of the transcription factor activating protein (AP-1) and enhanced promoter activity [39]. Another *TSLP* polymorphism investigated in the literature, rs10062929 (+591C/A), represents a variant located in the first intron of lfTSLP [31,32].

Given the nonspecific symptomatology and the lack of confirmatory diagnostic tools, there is an increased risk of both underdiagnosing and overdiagnosing non-IgE-mediated food allergy, underscoring the need to explore new markers that could act as risk factors and eventually aid in reaching a faster diagnosis. In the present study, we investigate for the first time the association of two specific *TSLP* SNPs (rs3806933 and rs10062929) with the risk of non-IgE-mediated food allergies and with different features of these patients.

## 2. Results

Demographic, anamnestic, clinical, and paraclinical characteristics of the patient group are described in Table 1. Girls were better represented than boys in our study group (58.6% vs. 41.3%), while an urban background was predominant (77.0%). The most frequent type of delivery was represented by cesarean section (66.6%). Regarding feeding practices, 80.4% of the children received breast milk for at least 14 days, but only 42.5% were exclusively breastfed. The median duration of breastfeeding was 6 months.

A positive family history of atopy (defined as the presence of an allergic disease in a parent or sibling) was reported in 47.1% of cases. In total, 27.5% of the children were diagnosed with atopic dermatitis, while elevated levels of total serum IgE were detected in 32.1% of the patients.

No significant difference was observed for sex distribution between controls and patients (*p* = 0.7).

The genotyping success rate was 100%, and the genotype distribution respected the Hardy–Weinberg equilibrium for both investigated SNPs in controls and patients.

The analysis of allele frequencies for the *TSLP* promoter SNP rs3806933 in the control group revealed that the minor allele was C in our population (44.8%). According to dbSNP, in the European population, the minor allele for this polymorphism is T, with frequencies of around 45% [40]. Data reported for the Estonian population (which is also Caucasian) in dbSNP show frequencies similar to our results, with allele C as the minor allele (47.7%). For the second analyzed SNP, rs10062929, the minor allele frequency in Romanian controls (18%) fell within the range reported for European ancestry populations in large-scale genetic datasets (13.4–22%) (dbSNP). Single-marker analysis showed similar allele and genotype frequencies between patients and controls for the two SNPs (Table 2).

Three haplotype combinations were present in the two groups (rs10062929/rs3806933 C/C, A/T, and C/T), with no significant difference in frequency between controls and patients (44.7%, 17.7%, 37.5%, and 43.2%, 18.3%, 38.4%, respectively). Linkage disequilibrium level was low (r^2^ = 0.14).

Following the analysis of rs3806933 in relation to different characteristics of the patients (gender, atopic dermatitis, family history of allergy, and total IgE levels), no significant results were obtained (Table 3). However, it was noted that the frequency of the minor allele C was lower in children with atopic dermatitis (35.4% vs. 46.8%), a positive family history of atopic disease (39.0% vs. 47.8%), and elevated levels of total IgE (39.2% vs. 45.7%).

When the allelic and genotype distributions of rs10062929 were analyzed in the subgroups of patients (Table 4), it was observed that the frequency of the minor allele A was lower in children who also presented atopic dermatitis (12.5% vs. 21.4%), but the difference was not statistically significant. Patients with a positive family history of allergy presented a higher frequency of the minor allele A (21.9% vs. 16.3%) and the AA + CA genotypes (39.0% vs. 28.2%), but the level of statistical significance was not reached. The frequency of the minor allele A was lower in children with high levels of total IgE compared to the patients with normal IgE values (14.2% vs. 21.1%), but this difference was not statistically significant.

## 3. Discussion

We report for the first time the investigation of *TSLP* gene polymorphisms and the broad category of non-IgE-mediated food allergies. The two analyzed SNPs were not associated with the risk of disease at the individual or haplotypic level.

In order to further explore other possible associations between the two investigated SNPs and specific clinical or paraclinical characteristics of the patients with non-IgE-mediated food allergy, a secondary aim of our study was to assess the correlations between the selected polymorphisms and the presence of atopic dermatitis, total IgE levels, and a positive family history of atopy. The patients were divided into two subgroups based on the total IgE values, based on data from the literature about the association between rs10062929 and the levels of total IgE [21]. The presence of atopic dermatitis was another characteristic we decided to focus on, due to data from the literature regarding the associations between *TSLP* polymorphisms and atopic dermatitis [21,30]. Furthermore, considering that a positive family history of atopy is largely recognized as one of the risk factors for developing food allergy and other types of atopic disease, we believe that exploring the association between the presence of a positive family history of allergy and the *TSLP* gene polymorphisms could provide valuable insight.

One of the polymorphisms we assessed in this study, rs3806933, was reported to be in linkage disequilibrium (LD) with other *TSLP* SNPs, including rs3806932, rs1898671, and rs1837253, in various populations [21,30,32]. Different studies demonstrated the association between rs3806932 and rs1898671 and the risk of eosinophilic esophagitis (EoE), which is one of the most-studied types of non-IgE-mediated food allergy [24,25,34,36]. Moreover, a series of studies, including animal models and humans, demonstrated the central role of TSLP in the development of EoE, as it drives multiple immune mechanisms that lead to specific histological changes [17,41]. The cases of EoE in our group were only a few (7 out of 87), so we could not test previous results reported in the literature regarding *TSLP* gene variation in EoE.

Rothenberg et al. performed a genome-wide association study to explore common genetic variants associated with EoE risk that included a discovery and replication cohort with a total of 351 EoE pediatric patients and 3100 controls, all of them having European ancestry [25]. The only genome-wide significant locus after multiple-testing correction was on chromosome 5q22, where the *TSLP* and *WDR36* (WD repeat domain 36) genes are located. The most significant SNP associated with EoE risk was rs3806932. The protective minor allele G was more frequent in controls than in patients with EoE (combined *p*-value = 3.19 × 10^−9^) [25]. Additionally, overexpression of *TSLP* was identified in the esophagus of EoE patients. Individuals with homozygous genotypes for the protective minor allele G of rs3806932 presented a lower *TSLP* expression (*p* < 0.0001). In the studied population, rs3806932 was in LD with 11 other SNPs that were also associated with EoE, including rs10062929 [25].

The association between the *TSLP* locus (including the rs3806932 polymorphism) and the risk of EoE was confirmed by another GWAS (N = 603 patients in the discovery cohort and 333 cases in the replication cohort, all of European ancestry) [34].

A study including adult EoE patients of Spanish European origin (N = 218) and healthy controls (N = 376) revealed that the minor allele A of rs10062929 was significantly less frequent in patients (OR = 0.68, *p* = 1.0 × 10^−5^) [36]. Furthermore, rs10062929 was associated with a lower risk of EoE when the dominant model was analyzed, comparing the frequencies of AA + AC vs. CC genotypes (OR = 0.23, *p* = 1.1 × 10^−6^) [36].

Another study assessing the association between several SNPs (from allergy and epithelial-relevant genes) and EoE was conducted by Sherril et al. and included a discovery cohort of 172 EoE patients with an allergic control group (N = 227), a non-allergic control group (N = 246), and a replication EoE cohort (N = 122) with a replication control cohort (N = 119), with all individuals being Americans with Caucasian origins [24]. The most important genetic variants reported to be associated with EoE risk were *TSLP* SNPs, and the association was independent of allergy [24]. The only statistically significant *TSLP* SNP after Bonferroni adjustments was rs10062929, only when EoE patients and allergic controls were compared (*p* = 4.11 × 10^−5^, OR = 0.35) [24].

Martin et al. evaluated the contributions of 372 single-nucleotide polymorphisms (SNPs) in 63 atopy genes to EoE development in Americans of European descent [42]. The study group comprised 700 EoE patients, while 412 non-atopic and 868 atopic disease control subjects were also enrolled. The EoE cohort partially overlaps (15%) with that included in the above-mentioned study conducted by Sherril et al. Associations between *TSLP* rs3806933 and the risk of EoE were described when both atopic (*p* = 0.00038, OR = 1.41) and non-atopic controls (*p* = 0.00018, OR = 1.34) were used. For *TSLP* rs10062929, the significant association with EoE risk persisted only in atopic controls after multiple-testing adjustment (*p* = 0.00047, OR = 1.50). Gene–gene interactions between *TSLP* and *IL4* genes SNPs were described (*p* = 0.0074), with children with risk alleles for both of the genes being at an increased risk of developing EoE (*p* = 2.0 × 10^−10^; OR, 3.67) [42].

The present analysis of the two *TSLP* SNPs in relation to different characteristics of the Romanian patients with non-IgE-mediated food allergies showed several differences regarding the presence/absence of atopic dermatitis, IgE level and a positive family history of allergy. However, they were not statistically significant given the limited number of patients in each subgroup.

In an American study including 309 children with EoE, no significant associations were identified between rs3806932 and the presence of atopic dermatitis, asthma, allergic rhinitis, IgE-mediated food allergy, or positive skin prick tests [35]. On the other hand, the investigated *TSLP* risk allele A (major allele) was significantly more frequent in children with multiple food allergen triggers (*p* < 0.0001). Furthermore, enhanced in vitro TSLP production by differentiated esophageal cells, induced by chicken egg ovalbumin, was observed in patients with a homozygous genotype for the risk allele compared to the heterozygotes (*p* = 0.03). Based on the lack of association between the *TSLP* risk allele and the presence of atopy in general or of a specific allergic condition, the authors hypothesized that the *TSLP* polymorphism might rather be correlated with the non-IgE-mediated food allergy immune reactions [35].

*TSLP* gene polymorphisms were also assessed in several other atopic pathologies, including atopic dermatitis, IgE-mediated food allergy, food sensitization, asthma, and allergic rhinitis, with the main findings being depicted below.

Regarding the risk of food sensitization, gene–environment interactions between *TSLP* rs3806933 and breastfeeding were observed (*p* = 0.006) in a prospective U.S. birth cohort study (N = 970) [43]. Children with genotypes including the minor allele T (CT + TT) of rs3806933 who were ever breastfed presented an increased risk for food sensitization (*p* = 0.0008), while the risk of food sensitization was not significantly different between ever breastfed and never breastfed in CC homozygous individuals (*p* = 0.777) [43]. Additionally, a longer duration of breastfeeding was also associated with a higher risk of food sensitization in children with rs3806933 CT and TT genotypes [43].

In a Polish study that included 109 atopic children (diagnosed with at least one of the following diseases: atopic dermatitis, IgE-mediated food allergy, and anaphylaxis), individuals carrying the *TSLP* SNP rs2289277 risk allele (C) presented a nearly double risk of developing an allergic condition, while the risk was nearly threefold higher in homozygotes for the C allele [37]. Children carrying the risk allele also presented a higher risk of having two or all three of the allergic diseases assessed (*p* = 0.009) [37]. The analyzed *TSLP* polymorphism was also correlated with the presence of sensitization to multiple allergens [37]. Children included in the study group presented higher levels of serum TSLP compared to the control group [37]. Furthermore, significantly higher serum TSLP levels were detected for patients with multiple allergic conditions [37]. No association between rs2289277 and TSLP levels was identified [37].

A study from the United States of America assessed the associations between several SNPs from the *TSLP, TSLPR*, and *IL7R* genes and atopic dermatitis and one of its complications, eczema herpeticum [21]. In both patient groups, one of European American origin (N = 444) and one of African American origin (N = 339), no significant associations were described for the rs10062929 SNP in relation to the risk of atopic dermatitis, the eczema area, and the severity index [21]. Another *TSLP* SNP, rs1898671, was strongly associated with a lower risk (*p* = 0.001) of eczema herpeticum in the European American patients, with this SNP being in strong LD with rs3806933 [21]. On the other hand, rs10062929 was significantly associated with total serum IgE levels in the European American group of patients (*p* = 0.031), but not in the group with African American ancestry [21]. The interaction between the *TSLP* rs10062929 SNP and *IL7R* rs11957503 was correlated with the risk of eczema herpeticum in patients with European American ancestry [21]. An association with atopic dermatitis was also reported for *TSLP* SNP rs1898671 in a study that included Americans of both European and African origin, in which it was also found that rs1898671 was not in LD with rs10062929 [44].

Miyake et al. explored the associations between three *TSLP* SNPs (rs3806933, rs1837253, and rs2289276) and eczema in Japanese women (N = 188) [30]. The results showed that rs1837253 was associated with the disease (*p* = 0.048), while no significant associations were identified in any of the genetic models analyzed between rs3806933 and rs2289276 and the risk of eczema [30].

Polymorphisms in the *TSLP* gene were also associated with asthma and allergic rhinitis [14,29,32,38,45,46]. Torgerson et al. conducted a meta-analysis of GWASs, including an ethnically heterogeneous American population that demonstrated an association between *TSLP* polymorphisms and asthma risk [47]. In another GWAS from Australia, SNPs in the *TSLP* gene were also linked to asthma susceptibility, but not to hay fever risk [48]. In a study from the Japanese population, rs3806933 was significantly associated with childhood atopic asthma (*p* = 0.0063) and adult asthma (*p* = 0.0023) [32]. The association between *TSLP* SNP rs1837253 and the reduced risk of asthma and allergic rhinitis has been reported by several studies [38,45,49,50,51,52].

All these results point to a certain influence of *TSLP* gene variations on the risk of allergic diseases. The extent of this influence remains to be determined in subsequent studies involving different populations and larger patient cohorts.

### Limitations

The main limitation of our study is the relatively modest sample size (87 patients and 115 controls) and, in particular, the small number of subjects within each clinical subgroup. In our cohort, the minor allele frequencies were approximately 45% for rs3806933 and 18–19% for rs10062929, which is in line with values reported for European populations. Under these conditions, the precision of several odds ratio estimates for both SNPs is limited, as reflected by the relatively wide confidence intervals, especially in the stratified analyses. Consequently, the non-significant findings reported here should not be interpreted as definitive evidence of the absence of association; rather, they should be viewed as hypothesis-generating and as a basis for designing larger, adequately sized studies.

Further research regarding specific genetic polymorphisms and non-IgE-mediated food allergy is necessary as it could provide valuable insights for future strategies regarding risk estimations, emerging diagnostic techniques, and targeted therapeutic interventions.

## 4. Materials and Methods

The study group included 87 pediatric patients diagnosed with non-IgE-mediated food allergy at Marie Curie Emergency Children’s Hospital, Bucharest, all of them being unrelated Caucasians of Romanian origin (36 males/51 females).

Seven children were diagnosed with EoE. The gold standard for the diagnosis of EoE was used: in each case, upper gastrointestinal endoscopy was performed with at least 6 biopsies from upper and lower levels of the esophagus (with at least two of the samples collected from the same level), and the histological examination of these samples revealed a peak eosinophil count of at least 15 eosinophils/high power field (HPF).

In 80 patients, the diagnosis of non-IgE-mediated food allergy was established based on the presence of a highly suggestive clinical history marked by consistent delayed reactions (such as failure to thrive, vomiting, chronic diarrhea, hematochezia, and chronic abdominal pain), with symptom reproducibility upon repeated exposure to the triggering food allergens, and the presence of a significant improvement or complete remission of the clinical manifestations after 1 month of respecting the food elimination diet.

In nine of the patients, significant improvement/remission of symptoms was not achieved after the 1-month initial elimination diet. In these cases, other food allergens were additionally excluded from the diet, and a second re-evaluation was performed after another month of the elimination diet, when remission or a significant improvement in the clinical manifestations was present.

Depending on the specific characteristics of each case, alternative diagnoses were ruled out based on clinical evaluation and laboratory results.

The diagnosis of atopic dermatitis was established in these patients in a consultation performed by a dermatology specialist at various times prior to inclusion in this study, depending on each specific personal history. The cutoff levels used for including the patients in the group with elevated total IgE were those provided by the Laboratory of the Marie Curie Emergency Children’s Hospital, where these blood tests were performed. The upper limit of the normal range varies with age, with the exact values being as follows: <15 IU/mL (0–12 months old), <60 IU/mL (1–6 years old), <90 IU/mL (6–10 years old), <200 IU/mL (10–15 years old), <100 IU/mL (>15 years old).

In the control group, 115 unrelated individuals of Romanian origin were enrolled (52 males/63 females). In each case, personal history was assessed in order to ensure the absence of any current or past allergic disease.

The approval of the Ethics Committee of Marie Curie Emergency Children’s Hospital was obtained, and all parents of the patients and controls provided informed consent to participate in the study.

Based on previous findings from the literature regarding the influence of *TSLP* gene SNPs on the risk of allergic disease and especially EoE [14,16,17,21,24,25,29,32,36,38,42,45,53], we selected rs3806933 (a promoter SNP with a certain impact on the level of transcription) and rs10062929 (an intron SNP).

Genomic DNA was extracted from peripheral venous blood using the QIAamp Blood Mini Kit (Qiagen, Hilden, Germany) according to the manufacturer’s protocol. We selected two SNPs from the *TSLP* gene for analysis, rs10062929 and rs3806933, that were genotyped using Real-Time polymerase chain reaction with TaqMan Allelic Discrimination Assays C_30152570_10 and C_3166722_10 (Real-Time PCR System, Applied Biosystems by Thermo Fisher Scientific, Foster City, CA, USA).

The statistical analysis of the data was performed using OpenEpi online software, version 3.01 (Atlanta, GA, United States) and PLINK v1.07 software (Cambridge, MA, USA). The level of significance was considered at *p* < 0.05. Demographic, anamnestic, clinical, and biological characteristics of the group of patients are reported as relative and absolute frequencies for categorical variables and as median and interquartile ranges for non-normally distributed continuous variables. Kolmogorov–Smirnov tests were performed in order to assess the normality of the continuous variables. The frequencies of alleles and genotypes were compared between the control and patient groups, and between specific patient subgroups, using Mid-P exact tests. The odds ratio and the 95% confidence interval were reported.

## 5. Conclusions

In this cohort of Romanian children with non-IgE-mediated food allergy, we did not detect any statistically significant associations between the *TSLP* polymorphisms rs3806933 and rs10062929 and disease status, nor with the presence of atopic dermatitis, a positive family history of atopy, or elevated total IgE levels. The observed allele and genotype distributions, together with the odds ratio and confidence interval estimates, provide the first characterization of these *TSLP* variants in this clinical phenotype and population. However, given the modest sample size and the limited precision of some estimates, particularly in subgroup analyses, these results should be interpreted with caution and considered hypothesis-generating. Future studies, including larger and phenotypically stratified cohorts, will be needed to confirm or refute potential weak associations and to further clarify the role of *TSLP* variation in non-IgE-mediated food allergy.

## Figures and Tables

**Table 1:** Demographic, anamnestic, clinical, and paraclinical characteristics of the patient group.

Characteristics	Patients (N = 87)
Gender M F	36 (41.3%) 51 (58.6%)
Age (median [IQR])	5 [1, 8] yo
Background Urban Rural	67 (77.0%) 20 (22.9%)
Cesarean delivery	58 (66.6%)
Ever breastfed	70 (80.4%)
Exclusively breastfed	37 (42.5%)
Duration of breastfeeding (median [IQR])	6 [3, 12] months
Positive family history of atopy	41 (47.1%)
Atopic dermatitis	24 (27.5%)
Elevated total IgE levels	28 (32.1%)
Pets at home	57 (65.5%)

**Table 2:** Results of the study of association of *TSLP* gene polymorphisms and non-IgE-mediated food allergy.

SNP	Controls (N = 115) N (%)	Patients (N = 87) N (%)	Statistics
rs3806933			
Minor allele			
C	103 (44.8%)	76 (43.6%)	OR = 0.95 95% CI 0.64–1.42 *p* = 0.82
Genotypes			
CC + CT TT	24 + 55 (68.7%) 36 (31.3%)	15 + 46 (70.1%) 26 (29.8%)	OR = 1.06 95% CI 0.58–1.95 *p* = 0.83
Heterozygotes			
CT TT + CC	55 (47.8%) 36 + 24 (52.1%)	46 (52.8%) 26 + 15 (47.1%)	OR = 1.22 95% CI 0.70–2.13 *p* = 0.48
rs10062929			
Minor allele			
A	41 (18%)	33 (19%)	OR = 1.07 95% CI 0.64–1.79 *p* = 0.76
Genotypes			
AA + CA	5 + 31 (31.3%)	4 + 25 (33.3%)	OR = 1.09 95% CI 0.60–1.98 *p* = 0.76
CC	79 (68.6%)	58 (66.6%)	
Heterozygotes			
CA AA + CC	31 (26.9%) 5 + 79 (73.0%)	25 (28.7%) 4 + 58 (71.2%)	OR = 1.09 95% CI 0.58–2.03 *p* = 0.78

**Table 3:** Allele and genotype distribution of rs3806933 for a series of characteristics of the patient group.

rs3806933	Minor Allele		Genotypes		
	C	Statistics	CC + CT	TT	Statistics
Gender M (N = 36) F (N = 51)	28 (38.8%) 48 (47.0%)	OR = 1.39 95% CI 0.75–2.57 *p* = 0.29	22 (83.3%) 39 (82.3%)	14 (16.6%) 12 (17.6%)	OR = 2.06 95% CI 0.81–5.24 *p* = 0.13
Atopic dermatitis No (N = 63) Yes (N = 24)	59 (46.8%) 17 (35.4%)	OR = 0.62 95% CI 0.31–1.23 *p* = 0.18	46 (73.0%) 15 (62.5%)	17 (26.9%) 9 (37.5%)	OR = 0.61 95% CI 0.22–1.66 *p* = 0.35
Allergy family history Negative (N = 46) Positive (N = 41)	44 (47.8%) 32 (39.0%)	OR = 0.69 95% CI 0.38–1.27 *p* = 0.24	33 (71.7%) 28 (68.2%)	13 (28.2%) 13 (31.7%)	OR = 0.84 95% CI 0.33–2.12 *p* = 0.73
Total IgE levels Normal (N = 59) High (N = 28)	54 (45.7%) 22 (39.2%)	OR = 0.76 95% CI 0.40–1.46 *p* = 0.42	43 (72.8%) 18 (64.2%)	16 (27.1%) 10 (35.7%)	OR = 0.66 95% CI 0.25–1.75 *p* = 0.42

**Table 4:** Allele and genotype distribution of rs10062929 for a series of characteristics of the patient group.

rs10062929	Minor Allele		Genotypes		
	A	Statistics	AA + CA	CC	Statistics
Gender M (N = 36) F (N = 51)	16 (22.2%) 17 (16.6%)	OR = 0.70 95% CI 0.32–1.49 *p* = 0.36	14 (38.8%) 15 (29.4%)	22 (61.1%) 36 (70.5%)	OR = 0.65 95% CI 0.26–1.61 *p* = 0.36
Atopic dermatitis No (N = 63) Yes (N = 24)	27 (21.4%) 6 (12.5%)	OR = 0.52 95% CI 0.20–1.36 *p* = 0.18	23 (36.5%) 6 (25.0%)	40 (63.4%) 18 (75.0%)	OR = 0.57 95% CI 0.20–1.66 *p* = 0.32
Allergy family history Negative (N = 46) Positive (N = 41)	15 (16.3%) 18 (21.9%)	OR = 1.44 95% CI 0.67–3.09 *p* = 0.35	13 (28.2%) 16 (39.0%)	33 (71.7%) 25 (60.9%)	OR = 1.62 95% CI 0.66–3.98 *p* = 0.30
Total IgE levels Normal (N = 59) High (N = 28)	25 (21.1%) 8 (14.2%)	OR = 0.62 95% CI 0.26–1.47 *p* = 0.28	22 (37.2%) 7 (25.0%)	37 (62.7%) 21 (75.0%)	OR = 0.56 95% CI 0.20–1.53 *p* = 0.26

## Data Availability

The data analyzed during the current study are not publicly available due to ethical considerations concerning sensitive clinical information from pediatric participants. Certain de-identified data supporting the findings may be made available upon reasonable request.

## Abbreviations

The following abbreviations are used in this manuscript:

SNP	Single-Nucleotide Polymorphism
TSLP	Thymic Stromal Lymphopoietin
EoE	Eosinophilic Esophagitis
IL	Interleukin
OR	Odds Ratio
CI	Confidence Interval
LD	Linkage Disequilibrium
